# Assessing the mineral accretion technique (MAT) for marine benthic restoration: a scoping review highlighting procedural weaknesses and evidence gaps

**DOI:** 10.7717/peerj.21440

**Published:** 2026-06-22

**Authors:** Gabriel Rivas-Mena, Ana Ruiz-Frau, Hilmar Hinz, Nikola Djordjevic, Mirko Djurovic, Vesna Mačić

**Affiliations:** 1 Institut za Biologiju Mora, University of Montenegro, Kotor, Montenegro; 2 Centro Ocenaográfico Illes Balears (COB-IEO, CSIC), Consejo Superior de Investigaciones Científicas, Palma, Balearic Islands, Spain; 3 Instituto Mediterráneo de Estudios Avanzados (IMEDEA), Consejo Superior de Investigaciones Científicas, Esporles, Spain

**Keywords:** Mineral accretion technique, Restoration, Benthic, Marine conservation, Artificial reef, Seawater electrolysis, Nature based solutions

## Abstract

**Background:**

Marine ecosystems are undergoing rapid degradation, highlighting the need for conservation and active restoration measures to halt and potentially reverse biodiversity and functional losses. The Mineral Accretion Technique (MAT), which combines artificial reef construction with seawater electrolysis, has recently gained interest as a restoration tool to enhance benthic recovery. However, the scientific evidence supporting its effectiveness remains limited and inconclusive. With the present scoping review, we aim to: (i) consolidate the existing scientific literature on MATs; (ii) analyze and evaluate experimental application of MATs as a benthic restoration tool; and (iii) synthesize current knowledge on it effects on benthic organisms’ health and recovery potential. Thereby, this scoping review serves as a pathway to popularize MATs among restoration academics and practitioners, as well as interdisciplinary professionals working in climate change mitigation, biodiversity loss or nature-based solutions.

**Methodology:**

This scoping review assessed the state of knowledge on MAT by conducting a structured bibliographic search using the PRISMA and Standards for Evidence Synthesis in Environmental Management guidelines on Scopus and Web of Science. The screening of the sources and their posterior data extraction were conducted in duplicate. The research landscape and patterns related to MAT terminology were analyzed using RStudio. Additionally, a quality assessment framework was developed and applied to evaluate the methodological robustness of the retrieved studies.

**Results:**

Of the 325 publications retrieved, only 33 were relevant. The majority of the body of scientific literature (82%) has been published in the last fifteen years. Nonetheless, the lack of consistent terminology has prevented MATs from being established as a benthic restoration technique. Despite 75.8% of studies claiming biological benefits, 90.1% of studies lacked adequate control groups, and over half failed to adequately report the results.

**Conclusions:**

We recommend the standardization of terminology, proposing “Mineral Accretion Technique (MAT)”, improved experimental design, and clearer reporting protocols. This review identifies key evidence gaps and provides a roadmap for establishing a robust and replicable research strategy for MAT. By addressing these crucial weaknesses, future research can effectively examine the ecological validity and restoration potential of MAT, enabling its integration into marine restoration strategies.

## Introduction

Marine ecosystems are degrading at an unprecedented rate, with over 60% of the oceans experiencing cumulative impacts from human activities (Halpern et al., 2015; Halpern et al., 2019). Conservation measures alone are insufficient to counteract the decline in biodiversity and ecosystem functioning (Duarte et al., 2020). Consequently, ecological restoration is gaining recognition as a tool to reestablish ecosystem structure worldwide (Duarte et al., 2020; Montseny et al., 2021). In support of these efforts, the United Nations (UN) has designated the period 2021–2030 as the “UN Decade on Ecosystem Restoration”, running in parallel with the “UN Decade of Ocean Science for Sustainable Development” to address the ongoing decline in ocean health (IOC, 2018; UN General Assembly, 2019).

Ecological restoration efforts aim to return degraded ecosystems to a recovery path that ensures the survival of their species and functioning while enabling adaptations to both local and global changes (Gann et al., 2018). Active ecological restoration seeks to accelerate recovery, especially in ecosystems dominated by slow-growing organisms that would otherwise require centuries to be restored naturally (Pandolfi & Jackson, 2006). This need is particularly true for benthic ecosystems, vulnerable habitats frequently formed by species with slow growth rates, which play a key role in supporting biodiversity and ecosystem services (FAO, 2016). However, innovative restoration techniques are needed to achieve scalable, successful, and cost-efficient outcomes (Danovaro et al., 2025).

In this context, restoration techniques such as transplantation or larval enhancement have been accompanied by the creation of artificial substrates in order to increase their efficiency (Abelson, 2006; Boström-Einarsson et al., 2020). These man-made substrates, known as artificial reefs, are designed to enhance the recruitment, growth, and survival of benthic organisms (Yanovski & Abelson, 2019). While the performance of artificial reefs is influenced by site-specific hydrodynamics and abiotic factors (Burt et al., 2009; Tanaya et al., 2025), substrate material represents a controllable variable that can be optimized for both shallow and deep-water benthic ecosystems (Perkol-Finkel, Shashar & Benayahu, 2006; Larcom et al., 2014; Girard, Lacharité & Metaxas, 2016; Lanham et al., 2025). Substrate rugosity, in particular, increases the available surface area, promoting higher colonization rates (Vivier et al., 2021; Lanham et al., 2025). Consequently, current research focuses on developing complex materials that mimic the rugosity, porosity, and chemical-mineral composition of natural rocky substrates and reefs (Margheritini et al., 2021; Bracho-Villavicencio, Matthews-Cascon & Rossi, 2023), which traditional materials used in the construction of artificial reefs such as concrete, tiles, plastic, metal or rubble failed to replicate. Herein, the low-voltage mineral deposited material arises as a potential substrate for benthic restoration, which can be produced only using the mineral accretion technique (Margheritini et al., 2021).

**Figure 1:**
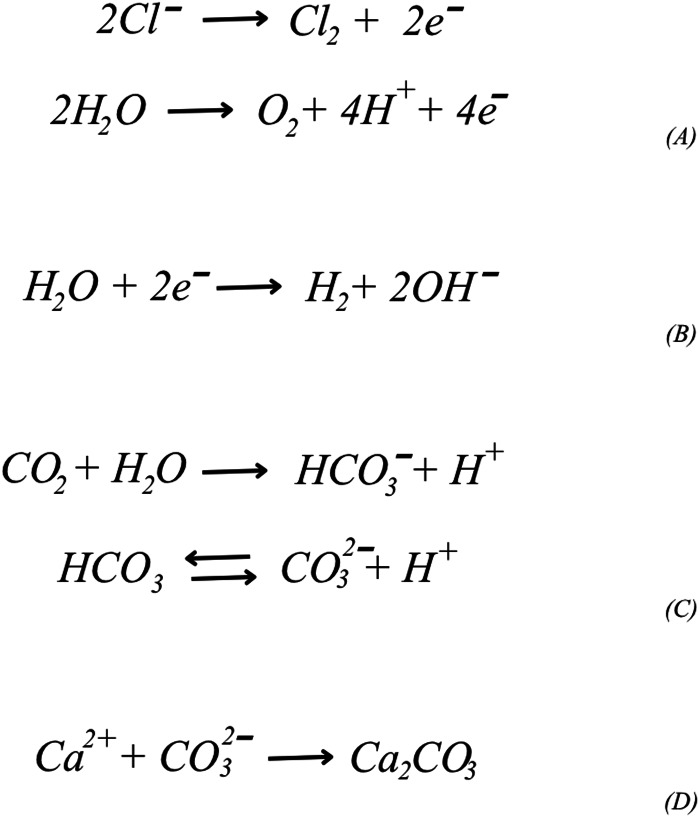
Reactions of the electrolysis process. (A) Reduction reaction in the cathode, (B) oxidation reaction in the anode, (C) natural seawater equilibrium, (D) precipitation of calcium carbonate due to the reaction of more carbonate ions near the cathode with free calcium ions in seawater.

The mineral accretion technique (MAT) was presented as an innovative method to enhance benthic recovery through seawater electrolysis (Hilbertz, 1979). This process involves an anode and a cathode through which electric power is applied to induce the precipitation of minerals from seawater (Hilbertz, 1979). The anode becomes positively charged, decreasing seawater pH and leading to the formation of Cl_2_ and O_2_ gases. Conversely, the seawater pH will increase in the cathode when it becomes negatively charged, inducing the formation of hydroxide (OH^−^) and H_2_ gas. The precipitation of calcium carbonate into the cathode is known as mineral accretion. Additionally, the hydroxide produced reacts with the carbon dioxide (CO_2_) dissolved in the seawater, producing bicarbonate (HCO^−^_3_) that subsequently reacts with the calcium (Ca^2+^) and Mg^2+^ already present in the seawater, thus precipitating calcium carbonate (CaCO_3_) at the surface of this electrode (Fig. 1). Additionally, the electrolysis of seawater could be used for carbon removal (La Plante et al., 2023); however, further research and advancement in engineering are needed to make MAT able to act as a carbon sink.

During mineral accretion, three different crystalline forms of calcium carbonate can be formed under standard seawater conditions: aragonite, brucite, and calcite (Hilbertz, 1979). The formation of each crystalline form varies depending on the alkalinity of the cathode, which might be determined by the salinity and temperature of the site. Nonetheless, this pH can be controlled with the optimal voltage calculated based on the environmental characteristics of the site (Barchiche et al., 2004; Margheritini et al., 2021). Seawater electrolysis resulting in a pH lower than 9.2 forms a mineral layer in the cathode’s surface with a major composition of aragonite. This is mostly achieved by lower voltages, thus the layer of mineral accretion formed is often denominated as Low Voltage Mineral Deposition (LVMD) material (Barchiche et al., 2004; Margheritini et al., 2021). In contrast, the increase of pH on the surface of the cathode over 9.2 due to an excessive power supply results in a major electrodeposition of brucite rather than aragonite (Barchiche et al., 2004), forming a layer of High Voltage Mineral Deposition (HVMD) material. Calcite is only formed at low rates since the Mg^2+^ from seawater inhibits its formation, favoring a major accretion of aragonite (Carré et al., 2020). In comparison to HVMD, the major composition of aragonite in LVMD characterizes this material with a chemical composition, density, total porosity, and pore-size distribution more similar to natural structures such as corallites (Margheritini et al., 2021).

Besides the creation of this complex substrate, MATs emerged as a restoration technique due to their capacity to increase the growth and survival of benthic organisms under stressful conditions by exposing them to electrotherapy (Goreau, 2022). The electrical field, in combination with the higher availability of calcium carbonate at the cathode, was thought to shift the energy invested in calcification to somatic growth and survival (Barchiche et al., 2004; Sokolova, 2021; Goreau, 2022). However these benefits on MATs in the life history traits of benthic organisms remain unsubstantiated. While some studies suggest that electrochemical conditions may enhance growth, survival, and larval recruitment (Kihara et al., 2013; Goreau, 2014; Goreau, 2022) by reducing calcification costs or improving resilience under stress, empirical evidence is inconsistent. Reported outcomes range from positive effects to negligible or even detrimental impacts on target organisms (*e.g.*, Strömberg, Lundälv & Goreau, 2010; Romatzki, 2014; Cook et al., 2023; Knoester et al., 2024). In the present study, we aim to provide a comprehensive assessment of the known effects of MATs on benthic recovery. Specifically, we evaluate parameters such as growth, survivorship, and larval settlement, alongside broader indicators of organismal health and associated community biodiversity.

Furthermore, research on MATs is limited and fragmented due to the lack of terminological consensus. Since the foundational work by Hilbertz (1979), this methodological intervention has been variously described using commercial, technical, and descriptive labels. It is frequently referred to as Biorock^TM^ (Goreau, Cervin & Pollina, 2004; Bakti et al., 2013; Berger et al., 2013; Fitri & Rachman, 2013; Goreau & Wolf, 2013; Jompa et al., 2013; Karissa et al., 2013; Nitzsche, 2013; Shorr et al., 2013; Vaccarella & Goreau, 2013; Zamani et al., 2013; Natasasmita, Wijayanti & Suryono, 2016; Munandar et al., 2018; Nugroho et al., 2023; Subhan et al., 2024), a commercial trademark. Other publications refer to it for its mineral accretion capacities as in the case of the terms: mineral accretion technique (Sabater & Yap, 2002; Sabater & Yap, 2004; Romatzki, 2014; Knoester et al., 2024), technology (Piazza et al., 2009; Borell, Romatzki & Ferse, 2010; Cook et al., 2023), or method (Strömberg, Lundälv & Goreau, 2010; Latchere et al., 2016). Only a few times it is named by its electrochemical properties, as is the case in the use of the terms electrochemical technique (Huang et al., 2020), or method (Kihara et al., 2013), electro-stimulated method (Samidon et al., 2022), and cathodically protected steel (Hunsucker et al., 2019; Hunsucker et al., 2021). Another more descriptive term that was used is the “in-situ formation of semi-artificial substrate by seawater electrolysis” (Van Treeck & Schuhmacher, 1997). Despite this semantic diversity, these terms all described the same benthic restoration technique developed by Hilbertz (1979), yet the lack of standardized nomenclature and differences in the voltage provided amongst studies hinder comparison across studies and limit replicability. To address this gap, we provide a comprehensive synthesis of the existing literature on MATs and their application in benthic restoration. Here, we refer to benthic restoration as the recovery of organisms or habitats, either sessile animals or plants, that dwell or colonize the sea bottom.

In this scoping review, we aim to: (i) consolidate the existing scientific literature on MATs; (ii) analyze and evaluate the experimental application of MATs as a benthic restoration tool; and (iii) synthesize current knowledge on their effects on benthic organism health and recovery potential. By clarifying both its limitations and opportunities, this scoping review encourages the advancement of restoration science by increasing the popularity of this technique among restoration academics and practitioners, ecological engineers, and policymakers, many of whom remain unaware of MAT’s potential applications. Furthermore, the intrinsic interdisciplinarity of MATs could offer new avenues for research in fields of knowledge exploring the effects of electrotherapy on marine organisms.

## Methods

A literature search was conducted, following the PRISMA guidelines (Tricco et al., 2018), on the 20th of March 2025 using the publication databases Scopus and Web of Science (WOS). Both databases were set to retrieve peer-reviewed articles, conference papers, and book chapters relevant to the study of MATs. The search string included terms related to benthic organisms and habitats (population), intervention (MAT), and outcome (restoration), as well as all the relevant synonyms used for these terms (*e.g.*, Biorock, Mineral Accretion Technique, Electric Artificial Reef). The final search string selected was constructed with the terms showing an optimal trade-off between the number of publications retrieved, relevant publications (precision), and sensitivity (the ability to retrieve as many relevant studies as possible) (Table S1). The search was performed across the title, abstract, and keywords of the publications (Text S1). Searches were restricted to English.

To complement this search and avoid missing relevant literature, a simplified search string (“Biorock” AND “Benthic” AND “Restoration”) was conducted in Google and Google Scholar. Following the Guidelines and Standards for Evidence Synthesis in Environmental Management (CEE, 2018), only the first 50 search results were reviewed.

**Figure 2:**
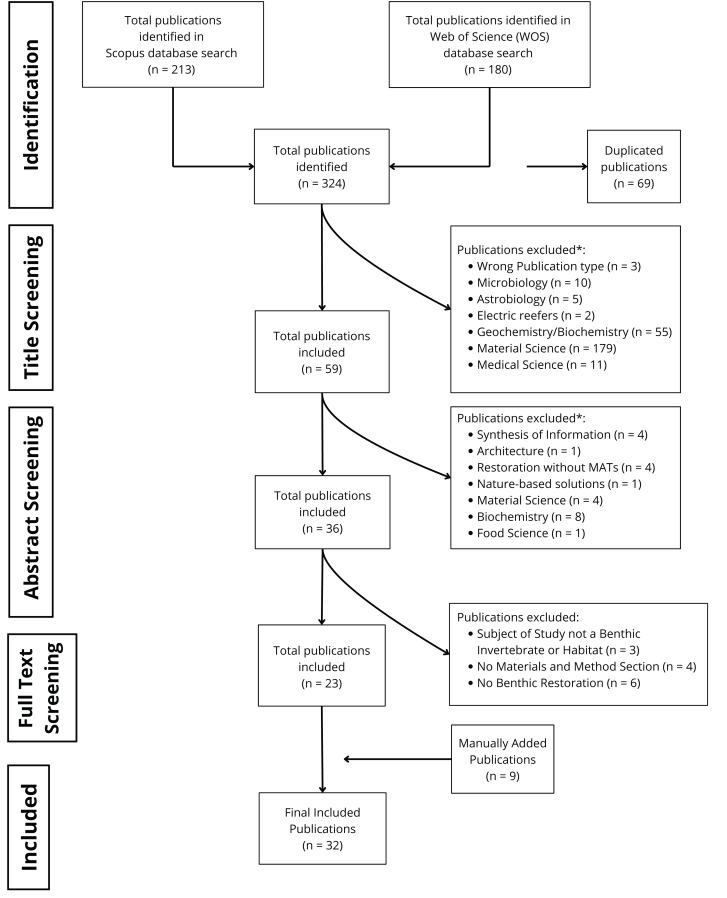
Scoping review PRISMA diagram. Selection process from publication retrieval to the ûnal analyzed literature. *During the title and abstract screenings, those publications not related to our research question were excluded. In the exclusion boxes are indicated the topics of excluded publications.

The initial search retrieved 213 publications from Scopus and 180 from WOS. No additional publications were retrieved from Google or Google Scholar. After removing duplicated sources (*n* = 69 publications), 324 publications were subjected to screenings conducted in duplicate by authors Gabriel Rivas-Mena (GRM) and Nikola Ðorđević (NÐ) in which the included sources were required to meet the following criteria: (i) population: a benthic organisms or habitat; (ii) intervention: recovery using the mineral accretion technique (MAT); and (iii) outcome: reporting at least one outcome related to the recovery of organisms or habitats. A first screening on sources title was conducted, followed by a second screening on their abstracts. Based on the two screening processes, 289 publications did not meet the inclusion criteria (Fig. 2). A third screening on the full text of the publication was conducted to ensure that all the sources followed the inclusion criteria and presented a Material and Methods section.

Following these criteria, 23 publications were selected. Additionally, the reference section of the included 23 research papers was revised to account for potential publications on MATs missed during the bibliographic database searches. Those relevant citations that contributed to addressing the objectives of this research were then included, increasing the final number of reviewed publications to 32.

The included sources were coded in two steps: (i) metadata extraction, in which title, authors, year of publication, journal, DOI, and keywords were recorded and assigned an identification code (ID); and (ii) content extraction, in which relevant information from the Material and Methods and Results sections was systematically extracted for each study (Table 1). The extracted Material and Methods data provided insights into study design, enabling the identification of potential confounding variables or methodological limitations such as absence of appropriate control groups or insufficient sample sizes. Similarly, the summary of the results facilitated the evaluation of MAT’s effect on fitness-related traits in benthic organisms and on the biodiversity of benthic habitats (Table S2). This data extraction was conducted in duplicate by author GRM and coauthor NÐ. Discrepancies in the data extraction from the sources were resolved through discussion or consultation with another reviewer.

**Table 1:** Coding of the extracted information from the publications.

**Category**	**Variable**	**Description**
Metadata	ID	Reference assigned to identify the publication
	Title	Publication title
	Authors	Name of authors
	Year	Year of publication
	Source	Name of journal, conference of book chapter
	DOI	Publication reference
	Keywords	Author’s keywords
Materials & Methods	Time	Amount of time of the experiment
	Subject of study	Organisms, community or benthic habitat
	Experimental design	Brief description of the experimental set up
	Control group	Description of the control group used to compare with treatment
	Treatment	Current density or voltage of the MAT
	Sample size	Number of samples studied
	Replication	Number of replicates used per treatment
	Sampling technique	Description of the sampling
Results	Fitness-related activities	Survival, Growth, Coverage, Branching, Budding, Reproduction, Fluorescence of studied organisms
	Biodiversity	Species richness, Shannon index, Simpson Index of studied communities or habitats

To analyze MAT’s research landscape and identify patterns and relationships between the terms related to MAT (*e.g.*, Biorock, mineral accretion, LVMD), a co-occurrence map of the keywords from the included publications was generated using R Studio (Rstudio team, 2020).

Following the Guidelines and Standards for Evidence Synthesis in Environmental Management (CEE, 2018) recommendations to assess the risk of bias of the study’s experimental design and results reporting, we adopted a quality assessment framework including the most relevant sources of bias for the use of MATs in benthic restoration (Table 2). This quality assessment framework aims to ensure both the methodological integrity of the revised papers and eliminate our potential bias while reviewing the publications by using a standardized evaluation criterion. The experimental design, timing of the experiment, inclusion of appropriate control groups, replicability, sample size, sampling technique, and result reporting of each study were evaluated using this framework.

**Table 2:** Quality assessment framework.

**Variable**	**Criteria**	**Score**
Experimental design	Control and treatments not separated (<10 m) or no control (Electric Field Influence)	0
	Clear separation between control and treatment, no possible influence (>10 m)	1
Timing	Unknown or inadequate duration of the experiment, no information provided on sampling period	0
	Adequate duration of the experiment with monitoring only before and after MAT implementation	1
	Adequate duration of the experiment with periodic monitoring	2
Control group	No control group or no specified control group	0
	Control group not valid due to clear different properties of the substrate material in comparison to low voltage mineral accreted material (materials like steel mesh, already existing communities, bamboo or concrete)	1
	Low voltage mineral accreted material (LVMD) or materials with similar properties (natural rock)	2
Current density of the treatment	Does not indicate any information about voltage or current density in the treatment	0
	Indicates Low or High voltage/current density but does not provide values	1
	Indicates exact values of the voltage/current density provided	2
Replication	No replication or unknown number of replicates or not enough replicates	0
	Replication with sufficient number of replicates	1
Sampling technique	Does not indicate any information about the sampling techniques employed	0
	Provides information about the sampling techniques employed	1
Results reporting	Does not provide any values just a description of the results or Information reported in the main text differs from figures, tables.	0
	Provides values of the measured outcomes, but does not report inferential statistics or significant differences, and does not provide exact measurements	1
	Provides values of the measured outcomes with inferential statistics	2

## Results

### Bibliometric analysis

Of the 324 publications retrieved from our searches, only 23 (7.1%) were relevant. In addition, nine publications of relevance were identified from the references of the selected papers and were added to the 23. Six of these literature sources were chapters from the book “Innovative Methods of Marine Ecosystem Restoration” (Goreau & Kent, 2013), and three were peer-reviewed articles not indexed in Scopus or WOS (Piazza et al., 2009; Natasasmita, Wijayanti & Suryono, 2016; Kihara et al., 2018). The 265 discarded publications belonged to fields of study unrelated to our research question (67.5% material science, 20.8% biogeochemical cycles, 11.7% other topics).

**Figure 3:**
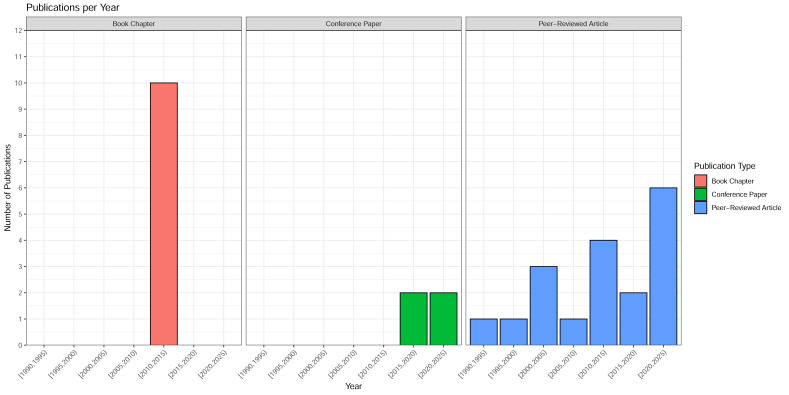
Number of published sources (book chapters, conference papers and peer-reviewed articles) on the Mineral Accretion Technique (MAT) for benthic restoration every five years. Colors indicate the source type.

The first publication exploring the effects of MATs on benthic organisms dates to 1994 (Schuhmacher & Schillak, 1994). Since then, 18 peer-reviewed articles, 10 book chapters, and four conference papers have been published (Fig. 3). The temporal analysis indicates a growing interest in this topic over the past fifteen years, with 82% of the literature published during this period. Moreover, there was a rise in both peer-reviewed articles (*n* = 12) and conference papers (*n* = 4) during this period of time.

**Figure 4:**
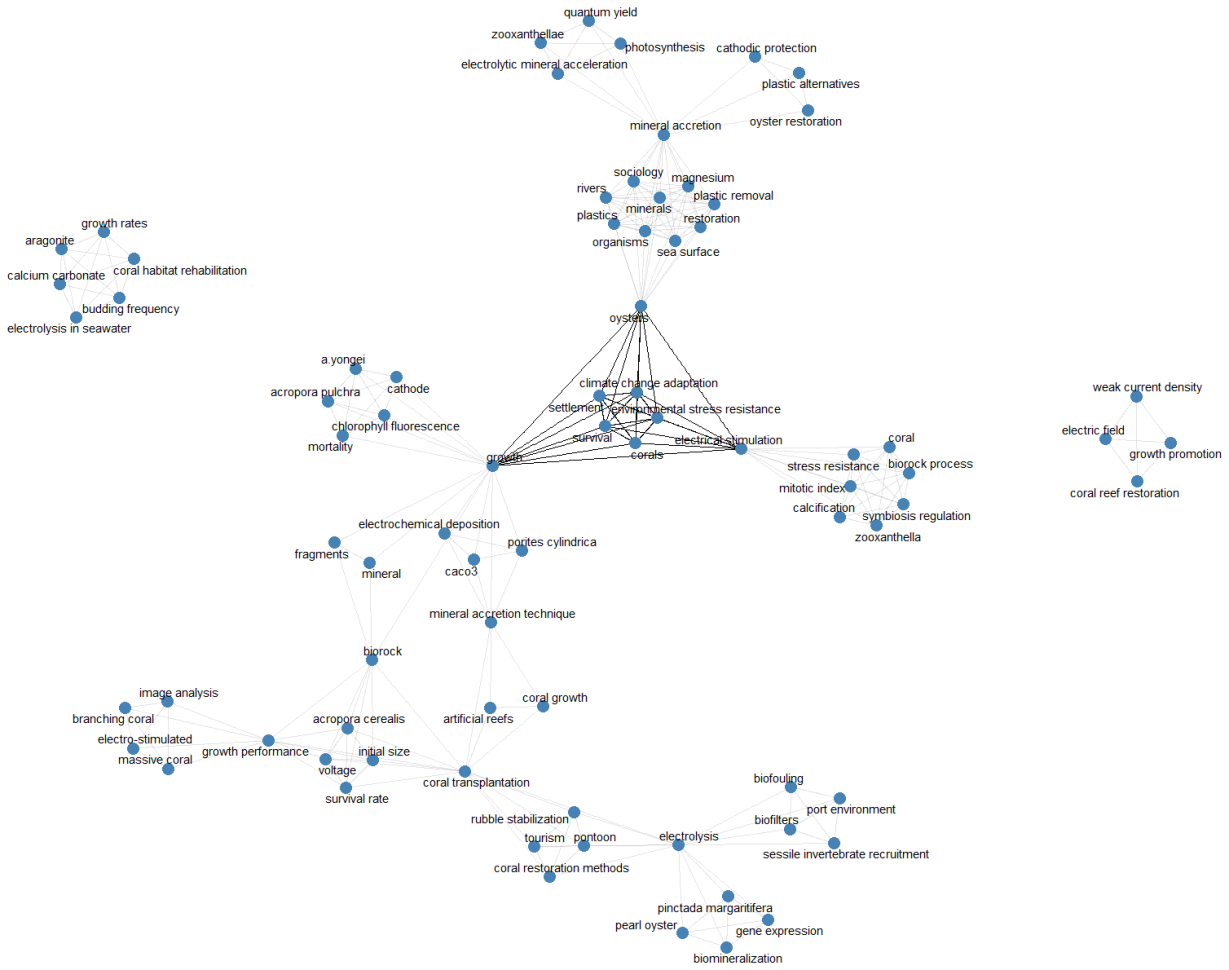
Co-occurrence map of the keywords from the included publications.

The co-occurrence map of keywords (Fig. 4) linked broad concepts (*e.g.*, “coral”, “survival”, “growth”) in a central cluster that diversifies into more specific terms. As a result, conceptually similar terms, such as “mineral accretion” (top left of the map) and “electrochemical deposition” (center of the map), appeared in unconnected groups of terms. The interchangeable use of terms to describe MATs is evident, indicating the lack of consistent terminology when referring to this restoration technique and the technical terminology around it (*e.g.*, “Seacrete”, “Seament”, or “Low Voltage Mineral Deposition Material”).

### Effects of MATs on benthic organisms

The majority of publications described MATs as benefitting the health of benthic organisms (75.0%, *n* = 24), either by affecting life history traits (*e.g.*, growth, survival, reproduction), physiological performance (*e.g.*, photosynthesis, gene expression) or ecological attributes (*e.g.*, increased in coverage, associated biodiversity), while the remaining publications reported either no effects or detrimental effects. Publications studied the effects on growth (65.6%, *n* = 21), survival (56.3%, *n* = 18), coverage (21.9%, *n* = 7), reproduction (9.4%, *n* = 3), photosynthesis performance (9.4%, *n* = 3), gene expression (3.1%, *n* = 1) and associated biodiversity (9.4%, *n* = 3).

Regarding the studied organisms, the majority of the studies were conducted on tropical coral species (65.6%, *n* = 21), while other organisms were underrepresented such as oysters (21.9%, *n* = 7), sponges (6.3%, *n* = 2), biofouling communities colonizing MATs (6.3%, *n* = 2), cold-water coral (3.1%, *n* = 1), and seagrass (3.1%, *n* = 1). 71.4% (*n* = 15) of the studies in tropical corals reported benefits on the growth (73.4%, *n* = 11), survival (53.4%, *n* = 8), coverage (20.0%, *n* = 3), fluorescence (13.4%, *n* = 2), reproduction (6.7%, *n* = 1), or associated biodiversity (6.7%, *n* = 1), although five of these studies did not report the *p*-values, confidence intervals, or effect sizes of their results. Regarding oysters, four studies (57.1%) reported benefits in their growth, three studies (42.9%) in their survival, two studies (28.6%) on their coverage, and one (14.3%) reported the upregulation of biomineralization-related genes, while another study did not report any benefit from the electrotherapy in oysters. Similarly, only 50% of the studies reporting benefits in oysters (*n* = 3) did report the complete statistical analysis. The publications exploring the effects of MATs on sponges, biofouling communities colonizing MATs, and seagrass, all reported benefits on the health of these organisms. Nonetheless, the only cold-water coral subjected to study, *Lophelia pertusa* (Linnaeus, 1758), did not exhibit any improvement in its growth rate or survival.

### Results of the quality assessment

The quality assessment revealed widespread methodological shortcomings across the reviewed studies (Table 3), particularly in spatial configuration, choice of control groups, reporting of treatment parameters, and statistical rigor. In the following sections, we detail the main limitations identified, highlighting how they may affect the reliability and interpretations of outcomes related to MAT effectiveness.

**Table 3:** Results of the quality assessment. Numbers represent the score provided following the quality assessment framework (Table 2).

Reference	Experimental design	Timing	Control group	Current density of the treatment	Replication	Sampling technique	Results reporting
Schuhmacher & Schillak (1994)	0	1	0	2	1	1	1
Van Treeck & Schuhmacher (1997)	0	2	0	2	1	1	1
Sabater & Yap (2002)	1	2	1	2	1	1	2
Goreau, Cervin & Pollina (2004)	0	1	1	1	0	1	0
Sabater & Yap (2004)	1	2	1	2	1	1	2
Piazza et al. (2009)	1	2	1	2	0	1	2
Borell, Romatzki & Ferse (2010)	0	2	1	2	1	1	1
Strömberg, Lundälv & Goreau (2010)	1	0	2	2	1	1	2
Bakti et al. (2013)	1	0	1	1	1	1	0
Berger et al. (2013)	1	2	1	2	1	1	1
Fitri & Rachman (2013)	0	2	1	2	1	1	2
Goreau & Wolf (2013)	0	0	0	2	0	0	1
Jompa et al. (2013)	0	2	1	2	0	0	2
Karissa et al. (2013)	0	2	1	0	1	1	2
Kihara et al. (2013)	0	2	1	2	1	1	1
Nitzsche (2013)	0	2	0	1	0	1	0
Shorr et al. (2013)	1	2	1	2	1	1	1
Vaccarella & Goreau (2013)	0	0	0	0	0	0	1
Zamani et al. (2013)	0	2	1	2	1	1	2
Romatzki (2014)	0	2	1	2	1	1	2
Latchere et al. (2016)	1	2	2	2	1	1	2
Natasasmita, Wijayanti & Suryono (2016)	0	2	1	2	1	1	1
Munandar et al. (2018)	0	2	1	2	1	1	1
Hunsucker et al. (2019)	0	2	2	2	1	1	1
Huang et al. (2020)	1	2	0	2	1	1	2
Hunsucker et al. (2021)	0	2	2	2	1	1	1
Samidon et al. (2022)	0	2	1	2	1	1	2
Carmona-Rodríguez et al. (2023)	1	1	1	0^*^	1	1	2
Cook et al. (2023)	1	2	2	2	1	1	2
Nugroho et al. (2023)	1	2	1	1	1	1	2
Knoester et al. (2024)	1	2	2	2	1	1	2
Subhan et al. (2024)	1	2	2	1	1	1	2

**Notes.**

* No electricity was applied for study purposes.

#### Spatial configuration

Adequate spatial configuration is important when experimenting with MATs, as the electrical field they generate can influence surrounding areas and potentially affect nearby controls. However, the reporting on the electrical field extension was inconsistent, and, in the majority of studies, no prevention measures were taken. This led to 56.3% (*n* = 18) of the revised publications setting their control group adjacent to the MAT, without considering the potential effects of the electrical field on them.

Only two publications attempted to empirically assess the extension of the electrical field range. Nitzsche (2013) reports that the electrical field’s influence diminishes beyond 3 m from the MAT, though these results should be interpreted with caution since 35 out of 66 measurements were removed from the analysis. Similarly, Bakti et al. (2013) deployed ten concrete blocks with sponges around a MAT, positioning five blocks to the north and five to the south, each spaced 2 m apart. This configuration resulted in a total distance of 10 m between the MAT and the outermost block in each direction. Despite reporting higher growth in the blocks near the MAT, their results are difficult to interpret since some of the sponges on the closest blocks exhibited lower growth rates than those located further away.

Some studies attempted to mitigate potential interference of the electrical field in untreated groups using spatial separation. In laboratory settings, Strömberg, Lundälv & Goreau (2010) used separate tanks for treatments and control groups. In field settings, separation distances ranged, *e.g.*, Knoester et al. (2024) maintained a 35 m distance, while Latchere et al. (2016) used a 20 m distance. Notably, many studies claimed separation between groups but did not report exact distances. No study systematically quantified the electrical field gradient *in situ* or under controlled conditions. Hence, the effectiveness of the spatial configuration remains unverified due to the lack of empirical measurements of field strength gradients.

#### Control groups

The majority of the studies used control groups with substrate materials that differed substantially from LVMD or natural rocks (81.3%, *n* = 26). Among these, four studies lacked a control group entirely, and two mentioned the existence of control groups without specifying the materials used. Materials with substantially different substrate properties to LVMD or natural rocks were frequently used for the control groups, such as uncoated steel frames of the cathodes (*n* = 12), pre-existing reef communities (*n* = 5), concrete (*n* = 2), bamboo (*n* = 1), or ceramic tiles (*n* = 1).

In a few cases, multiple control materials were tested within a single study. For example, Romatzki (2014) conducted parallel experiments using uncoated steel and bamboo as controls, while Kihara et al. (2018) compared uncoated steel and ceramic tiles in two distinct experiments. As a result, 22 control substrates were used across 20 studies.

Seven studies were not included in the above count, either because their control group appropriately aligned with the study’s objective or employed LVMD or natural rock. For example, Hunsucker et al. (2019) and Hunsucker et al. (2021) compared plastic mats with LVMD to assess substrate-specific effects, while Subhan et al. (2024) evaluated long-term reef development by comparing sites restored with MATs to adjacent reefs with no MAT intervention, both restored a decade earlier. Unlike other studies that compared MATs to pre-existing ecosystems, this design accounted for restoration age. Some studies made efforts to control for substrate effects. Strömberg, Lundälv & Goreau (2010) included both an uncoated steel mesh control and a procedural control using LVMD, accounting for the possible differences between the substrate of the control group and the treatments. Similarly, Knoester et al. (2024) and Latchere et al. (2016) preconditioned the control substrates by allowing a layer of LVMD to form on the cathode one month prior to the experiment. Cook et al. (2023) used natural rocks as controls but placed them adjacent to MATs, introducing a potential bias through spatial configuration and the aforementioned interference of the electrical field.

#### Duration, replication, and sampling

Compared to other methodological constraints, shortcomings related to the duration of the experiment, replication, and sampling were less frequent but still notable. The average duration of MAT experiments was 488 days (approximately 16 months). Subhan et al. (2024) assessed community composition with visual transects conducted over just two days, since their study area was restored using MATs a decade earlier. In contrast, Goreau & Wolf (2013) ran their experiment for eight years. Four studies (12.5%) did not run the experiment for enough time or did not specify the timing at which measurements were conducted. Designing the experiment’s duration requires accounting for species life-history traits. For example, Huang et al. (2020) and Fitri & Rachman (2013) limited their experiments to two months because they focused on fast-growing corals. Conversely, a six-month duration may have been insufficient to detect differences in the slow-growing cold-water coral *L. pertusa*. Although Strömberg, Lundälv & Goreau (2010) did not find significant differences between the lowest-voltage treatment and the control, they still observed that growth under low-voltage electrotherapy exceeded control growth by 0.074 mm/year by the end of the experiment. Periodic sampling is vital to detect when MATs begin to generate measurable effects. For instance, Latchere et al. (2016) identified the exact week when pearl oysters in the treatment group exhibited significantly higher growth than the control, avoiding premature termination of the experiment before MAT effects became evident.

Regarding replicability and sampling, among the 32 studies reviewed, 18.8% (*n* = 6) did not specify their number of replicates or based their measurements on fewer than three measurements per treatment group (Hurlbert, 1984; McLachlan et al., 2020), thereby limiting the robustness of their conclusions. Additionally, 9.4% (*n* = 3) failed to report the sampling techniques used to measure their variables.

#### Applied current densities

Applied current densities varied across studies, often compromising experimental replicability. Specifically, three studies (9.4%) did not report any current values used, while four (12.5%) provided only approximate voltage categories (*e.g.*, low, medium, or high voltage) without quantitative values. Among the remaining studies, reported voltages ranged from 1 V to 120 V, and amperages from 0.01 A to 12 A.

Inconsistencies were also evident in how treatment parameters were reported. Some studies provided both total voltage and amperage (*n* = 11), others reported only one of these values (*n* = 8), and a few provided values standardized by area (*e.g.*, per m^2^) (*n* = 3). Notably, only 2 studies reported both the amperages per m^2^ and total voltage. Moreover, Carmona-Rodríguez et al. (2023) did not report current values, as electricity was used solely to generate the LVMD layer before deployment of the MAT structure.

#### Statistical reporting

Differences in statistical reporting were observed in half of the reviewed studies. Specifically, 9.4% (*n* = 3) presented only descriptive outcomes without any quantitative data, and 40.6% (*n* = 13) failed to report full statistical test results, such as *p*-values, confidence intervals, or effect sizes.

In several instances, discrepancies were identified between the conclusions stated in the text and the data presented in figures and tables. For example, Bakti et al. (2013) concluded that sponge growth was reduced with distance from the MAT and that there were no differences between north and south placement. Yet, their graph showed higher growth rates on the southern blocks farther from the structure. Similarly, Strömberg, Lundälv & Goreau (2010) concluded that the lowest current density tested (0.06 A m^−2^) had a positive effect on *L. pertusa*, despite reporting no statistically significant differences compared to the procedural control group or a 0.4 V galvanic current. These inconsistencies suggest instances of selective interpretation that may overstate the effectiveness of MATs.

## Discussion

### MATs in the scientific literature

Scientific literature on MATs remains scarce, as shown by our results. Although our search strategy was highly sensitive, retrieving all publications indexed in scientific databases, only 7.1% of the retrieved studies were directly related to our research question, indicating low precision. This low precision stems from the inconsistent use of terminology across disciplines and the absence of a standardized definition for “Mineral Accretion Technique (MAT)”. Terms such as Biorock or Electric Artificial Reefs are often used interchangeably to describe MATs. Additionally, specific vocabulary related to MATs has not been established in the restoration literature, requiring the inclusion of multiple overlapping terms during the search. Terms like “Seacrete”, “Seament”, and occasionally “Biorock”, refer to the Low Voltage Mineral Deposition (LVMD) material formed through seawater electrolysis, while also having distinct meanings in fields such as material science (Ben-Nissan, 2003; Meyers et al., 2011).

A key barrier to the scientific advancement and broader recognition of MATs is the absence of standardized terminology and methodological protocols. This inconsistency prevents the visibility of relevant studies in scientific databases and impedes cross-study comparisons and replication. As a result, it limits the consolidation of knowledge and the development of robust scientific evidence needed to evaluate the effectiveness and applicability of MAT in marine ecosystem restoration. Hilbertz & Goreau (1996), the developers of the technique, originally described it as a “method of enhancing the growth of aquatic organisms, and structures created thereby”. They later introduced the term “Biorock^TM^” as a registered trademark to refer to this technology (Global Coral Reef Alliance, 2009), which subsequently gained widespread use. However, scientific publication guidelines (APA, 2020; Clarivate, 2025; Elsevier, 2025) discourage the use of trademarks in scientific and technical terminology. This has contributed to the heterogeneity of keywords found in the literature, with the second most used term referring to this technology as “MAT” (Sabater & Yap, 2002; Sabater & Yap, 2004; Romatzki, 2014; Knoester et al., 2024).

The increasing interest in this restoration technique over the past fifteen years highlights the need for consistent terminology to support the integration and visibility of future research. To address this, we propose adopting “Mineral Accretion Technique (MAT)” as the standardized term to describe the benthic restoration method that involves creating artificial reefs through seawater electrolysis that precipitates minerals such as aragonite and brucite onto the cathode’s surface, forming a layer of “Low Voltage Mineral Deposition (LVMD) material” that serves as the substrate, while simultaneously generating an electrical field. The term MAT would encompass the aforementioned terms referring to the technology developed by Hilbertz (1979), providing a neutral, descriptive alternative that can improve indexing in scientific databases and support the systematic accumulation of knowledge in marine ecosystem restoration.

### Current knowledge

MATs were initially proposed as a method to enhance the health of marine organisms by creating artificial structures subjected to electrical fields, accelerating ecological restoration (Hilbertz & Goreau, 1996). The original developers of this technique attributed the observed benefits to the electrotherapy properties of MATs. Indeed, 75.0% of the analyzed literature reported beneficial effects on organisms after MAT intervention. According to Goreau (2022), such enhancement occurs due to the reallocation of adenosine triphosphate (ATP) and nicotinamide adenine nucleotide phosphate (NADP) from maintenance processes to fitness-related activities (*e.g.*, survival, growth, reproduction), reducing environmental stress. However, this physiological mechanism remains hypothetical, as no studies have directly examined ATP reallocation or other metabolic energy pathways in organisms exposed to MATs. The assumption is instead based on broader stress physiology principles, which suggest that environmental stressors can shift the allocation of ATP away from homeostatic set points (Kültz, 2020; Sokolova, 2021).

Empirical evidence supporting these physiological claims is limited. Only one study (Latchere et al., 2016) investigated the effects of MATs on the gene expression of biomineralization-related genes, finding that electrography induces the upregulation of these genes in the oyster *Pinctada margaritifera* (Linnaeus, 1758). Conversely, Knoester et al. (2024) reported negative effects on four coral species exposed to MATs during a marine heatwave, contradicting the hypothesis that MATs enhance the reallocation of ATP from maintenance to fitness-related processes under stressful conditions. However, the authors acknowledged that, despite using current values recommended by the original developers of MAT, the electrical intensity applied might have been excessively high, causing such detrimental effects. They further noted that inconsistent reporting across studies made it impossible to determine whether their current density values were appropriate. In addition, the limited representation of taxa from temperate and cold-water systems, along with low overall taxonomic diversity, further constrains the understanding of the physiological effects of MATs. Another aspect often underrepresented is the effects of the electrical field on benthic-associated fauna, such as fish or elasmobranchs. Goreau (2022) described the presence of fish, sea turtles, and bottom-feeding elasmobranchs, while Uchoa, O’Connell & Goreau (2017) observed changes in the behavior of two predatory shark species around the MAT due to their electroreception.

Therefore, the effects of the electrotherapy induced by MATs on the health of benthic organisms remain unclear. Despite the increased research during the past fifteen years, the persistent uncertainty regarding the effectiveness of MAT is preventing it from becoming a widely recognized restoration technique. Our quality assessment indicates that this lack of evidence is a byproduct of systemic methodological shortcomings. In the following sections, we examine the methodological limitations identified in the reviewed studies to better understand the sources of such ambiguity and provide a series of standards for MAT research.

### Lack of proper control groups

Our quality assessment revealed that 54.5% of the studies did not consider the potential influence of the electrical field on control groups, and 81.3% employed control groups that were inappropriate for isolating treatment effects. In experimental designs, control groups must be comparable to treatment conditions, in all aspects except the variable being tested, *i.e.,* the electrical field, to enable valid inference (Underwood, 1990; Elphick, 2008). The consistent failure to meet this criterion in MAT studies introduces systematic bias in the reported outcomes that has contributed to the uncertainty on the effects of electrotherapy on benthic organisms.

A key complication is the limited understanding of the spatial extent of the electrical field generated by MATs. To date, no study has empirically characterized the full extent of the field generated by MATs. Despite this, clear spatial separation between treatments and controls is essential to minimize potential interference in control groups. A few studies have addressed this issue by maintaining sufficient distances or using separate tanks (Strömberg, Lundälv & Goreau, 2010; Latchere et al., 2016; Knoester et al., 2024), but most did not report separation distances or failed to isolate the control groups effectively.

Equally important in experimental design is the physical nature of the control substrate. Since MATs inherently produce LVMD, this material forms the natural basis for benthic colonization in all MAT treatments. LVMD exhibits mechanical, thermal, and hygroscopic properties similar to concrete (Johra et al., 2021), a material widely used in artificial reef construction (Bracho-Villavicencio, Matthews-Cascon & Rossi, 2023). Additionally, Margheritini et al. (2021) demonstrated that LVMD offers a more uniform pore size distribution compared to coral porite exoskeletons (a biogenic material formed by coral colony remnants), while maintaining a chemical composition similar to natural substrates. These properties make LVMD more comparable to natural rocks than concrete (Margheritini et al., 2021). Given the critical role of substrate properties in shaping benthic community development (Ushiama et al., 2016; Fong et al., 2024), control groups must be established on substrates with equivalent characteristics. Consequently, to accurately isolate the effects of electrical fields, control groups should be established on LVMD or natural rock structures. When using LVMD, it is advisable to precondition the control substrate by allowing mineral deposition to accumulate on the cathode for one month prior to initiating the experiment (Latchere et al., 2016; Knoester et al., 2024).

Of the reviewed studies, only three used control groups that met these methodological standards (Strömberg, Lundälv & Goreau, 2010; Latchere et al., 2016; Knoester et al., 2024). In contrast, others employed dissimilar materials such as uncoated steel frames, which some authors have criticized for introducing confounding factors, particularly corrosion and substrate properties that differ markedly from LVMD or natural rocks (Cook et al., 2023; Knoester et al., 2024). Regarding the use of already existing reefs as control groups, Jompa et al. (2013) reported faster growth in transplanted fragments of *Acropora nobilis* (Dana, 1846) on MAT structures compared to nearby natural reefs. However, this difference may reflect control group bias rather than the effect of electrotherapy. Several studies have reported faster growth rates in coral fragments than in established colonies (Forsman et al., 2015; Page, Muller & Vaughan, 2018; Schlecker et al., 2022), suggesting that comparisons with established reef communities can confound interpretations of treatment effects. Additionally, the structure of natural reefs might differ considerably from the deployed MAT structures, introducing new confounding variables when testing for the effects of electrotherapy (Perkol-Finkel, Shashar & Benayahu, 2006).

Consequently, the widespread use of inappropriate control groups for their spatial configuration or substrate material accounts for 90.6% of the reviewed studies, undermining the reliability of their conclusions and contributing significantly to the uncertainty surrounding MAT effectiveness.

### Methodological replicability of the treatments

Experimental designs must facilitate replicability (Casler, 2015). Detailed reporting of the experimental design, including sample size, duration, replication, and sampling techniques, is critical to enable experimental replicability. However, the majority of the publications on MATs lacked key methodological details: 12.5% ran their experiment for an insufficient amount of time or did not report when they conducted their samplings, 18.8% did not include adequate replication, 9.4% failed to describe their sampling techniques, and a majority reported treatment parameters inconsistently. This variability limits the reproducibility of findings and complicates efforts to compare outcomes across studies.

A major source of inconsistency lies in the electrical parameters applied. Voltage and current values varied widely, with several studies providing only approximate descriptions or omitting key measurements such as current density per unit area. Knoester et al. (2024), for example, reported the impossibility of determining whether their applied current density was appropriate due to the lack of reference values in prior research. Additionally, the effectiveness of MAT treatments is not only influenced by electrical settings but also by the environmental conditions and context of the study site, since factors such as salinity, temperature, and depth influence the electrolysis of seawater and mineral deposition rates.

In addition to environmental variability, species-specific responses to electrical stimulation may further complicate standardization. Organisms within the same genus have shown divergent responses to MATs (Sabater & Yap, 2002; Romatzki, 2014; Knoester et al., 2024), highlighting the need to account for taxon-level variation when designing experiments and selecting treatment parameters.

The original developers of MATs recommended applying voltages above 1.2 V, ideally between 6 and 12, within a safe range of 3 to 15 V, and current densities between 0.1 and 30 A m^−2^, in habitats with temperatures between 12 to 30 °C (Hilbertz & Goreau, 1996). Adequate mineral deposition should result in growth rates of 1 to two cm year^−1^ and a mineral composition of approximately 80.5% aragonite, 19.0% brucite, and 0.5% calcite (Goreau, 2012; Margheritini et al., 2021). Deviations in electric power supply can lead to suboptimal mineral structures, which can hinder benthic organisms’ development (Margheritini et al., 2021). Still, several studies attributed detrimental effects on their organisms to an excessive current density despite using values lower than the recommended (Hilbertz & Goreau, 1996; Strömberg, Lundälv & Goreau, 2010; Benedetti et al., 2011; Knoester et al., 2024). These findings suggest that optimal electrical settings must be tailored not only to the environment but also to species-specific sensitivities.

To improve replicability, future studies should report complete electrical parameters (*i.e.,* voltage and amperage per unit area) along with the environmental characteristics of the study site. This will enable cross-study comparisons and support the development of standardized treatment protocols tailored to both habitat conditions and species.

### Deficiencies in statistical reporting

More than half of the studies reviewed did not meet basic standards for result reporting. Specifically, few studies presented only descriptive outcomes without any accompanying measurements (9.4%), while almost half failed to report essential statistical details such as *p*-values, confidence intervals, or effect sizes (40.6%). These omissions contravene widely accepted publication guidelines (APA, 2020; Clarivate, 2025; Elsevier, 2025) and undermine the interpretability and scientific value of the results.

In some cases, reported conclusions contradicted the data presented in figures and tables, raising concerns about selective interpretation. Studies should include complete statistical outputs, including effect sizes, confidence intervals, and clearly labeled figures and tables, to provide an accurate reflection of the reality of the study’s findings. In addition, journals should encourage the publication of null or negative findings to avoid reinforcing biased perceptions about the efficacy of MATs, as these contribute to a more accurate and comprehensive understanding of MAT effectiveness and limitations.

### Costs and scalability of using MATs for marine restoration

Costs remain the primary constraint on marine restoration efforts (Bullock et al., 2011), ranging from approximately 0.5 to 25 million USD per hectare for different techniques (Bayraktarov et al., 2016). To mitigate these limitations, several benthic restoration methodologies have been developed (Kimball et al., 2015; Montseny et al., 2020). Within this context, MATs represent a promising yet still nascent approach to artificial reef construction, combining electrochemical precipitation with marine ecosystem enhancement.

One of MAT’s most compelling advantages lies in its theoretical potential to sequester CO_2_. During mineral accretion, the formation of calcium carbonate makes the surrounding waters more acidic, increasing the partial pressure of CO_2_ (Frankignoulle, Canon & Gattuso, 1994). Nonetheless, La Plante et al. (2023) demonstrated that net carbon dioxide removal is achievable during seawater electrolysis if the system achieves a hydroxide production at the cathode and the acid neutralization at the anode, since the hydroxide will react with the CO_2_ dissolved in seawater. Technologies capable of neutralizing acidity already exist such as bipolar membrane electrodialysis (Tongwen, 2002) or direct seawater electrolysis (Yu et al., 2025). Yet, this reality is still far away, further research and advancement in engineering are needed to better understand what are the real climate mitigation capacities of MATs.

Although MATs must overcome key aforementioned scientific hurdles, the formation of a LVMD layer may provide a cost-effective alternative capable of competing with land-based artificial reef development. The cathodes can be shaped to fit specific purposes before initiating the precipitation process. The main costs are associated with the anode, the installation of a suitable electricity power source, and the steel used for cathode construction. Importantly, anodes can last over a century under low voltage and, being resistant to corrosion during seawater electrolysis, enable the development of multiple LVMD reefs from a single anode. Still, constraints related to the need for underwater infrastructure, such as cables and a stable energy supply, may limit their scalability and increase their costs. Additionally, the ecological impact of sustained electrical fields remains poorly understood. Potential alterations in faunal behavior and the long-term consequences on marine biodiversity require further investigation.

While still in an early stage of development, MAT holds promise as an innovative solution at the intersection of marine conservation and climate technology, provided that its technical, ecological, and economic challenges can be systematically addressed.

## Conclusions

A total of 32 publications investigating the application of the Mineral Accretion Technique (MAT) were identified using the PRISMA guidelines. Despite the first publication using MATs as a restoration method dating back to 1994, this technique has not gained much attention until recently. Its limited uptake can be attributed to conceptual ambiguity, methodological inconsistency and poor replicability. To address these barriers, we propose reinforcing the use of the term “Mineral Accretion Technique (MAT)” as a neutral, standardized term. This standardization will facilitate consistent indexing in scientific databases and improve the integration of future research into marine restoration literature.

Although 75.0% of the studies reported beneficial effects on benthic organisms, such results should be interpreted with caution. The reviewed literature revealed substantial methodological shortcomings: the majority used inadequate control groups (90.6%), failed to ensure experimental replicability (34.4%), and did not report statistical outcomes in line with scientific standards (53.1%). These limitations undermine the strength of current knowledge about MAT effectiveness and highlight the need for more rigorous experimental designs in order to overcome the current data scarcity and yield robust evidence for this restoration technique.

To advance this field, we propose several key recommendations. First, future studies should adopt clear and consistent terminology and provide full documentation of treatment parameters, including voltage, current density per unit area, and site-specific environmental conditions. Second, appropriate control groups, ideally using natural rock or preconditioned LVMD, must be employed and spatially separated from treatment groups to avoid confounding effects from the electrical field. Third, studies should report all statistical results transparently, including null and negative outcomes, and align textual conclusions with presented data. Finally, given that species- and environment-specific factors strongly influence treatment outcomes, searches should be tailored to specific taxa and ecological context, to clarify under which conditions MATs are most effective and for which organisms.

While the overall benefits of the electrical field generated by MATs on benthic organisms remain unclear, the material properties associated with the formation of a low-voltage mineral deposited (LVMD) layer offer promising opportunities for the construction of artificial reefs. These structures can provide cost-effective, scalable substrates for ecological restoration. Further research should evaluate the long-term ecological performance of MATs across a range of environments and taxa supported by standardized and reproducible methodologies.

##  Supplemental Information

10.7717/peerj.21440/supp-1 Supplemental Information 1 Search string selection

10.7717/peerj.21440/supp-2 Supplemental Information 2 Data

10.7717/peerj.21440/supp-3 Supplemental Information 3 PRISMA checklist Extension for Scoping Reviews

10.7717/peerj.21440/supp-4 Supplemental Information 4 Data extracted from the selected publications

10.7717/peerj.21440/supp-5 Supplemental Information 5 Selected search string

## Additional Information and Declarations

### Data Availability

The following information was supplied regarding data availability: The data is available in the Supplemental File and at Zenodo: Rivas-Mena, G. (2025). Assessing the mineral accretion technique (MAT) for marine restoration: systematic review highlighting procedural weakness and evidence gaps_Rivas-Mena_ResearchData [Data set]. Zenodo. https://doi.org/10.5281/zenodo.17278118.
