# Supplementary material for: Assessing the mineral accretion technique (MAT) for marine benthic restoration: a scoping review highlighting procedural weaknesses and evidence gaps
Source: PeerJ. 2026 Jun 22;14:e21440. doi: 10.7717/peerj.21440 (PMC13296807; doi:10.7717/peerj.21440)
Supplement: Supplemental Information 3 [file peerj-14-21440-s003.docx]

**Preferred Reporting Items for Systematic reviews and Meta-Analyses extension for Scoping Reviews (PRISMA-ScR) Checklist**

| **SECTION** | **ITEM** | **PRISMA-ScR CHECKLIST ITEM** | **REPORTED ON PAGE #** |
| --- | --- | --- | --- |
| **TITLE** | | | |
| Title | 1 | Identify the report as a scoping review. | Line 2, page 1 |
| **ABSTRACT** | | | |
| Structured summary | 2 | Provide a structured summary that includes (as applicable): background, objectives, eligibility criteria, sources of evidence, charting methods, results, and conclusions that relate to the review questions and objectives. | Line 21 – 49, page 1 |
| **INTRODUCTION** | | | |
| Rationale | 3 | Describe the rationale for the review in the context of what is already known. Explain why the review questions/objectives lend themselves to a scoping review approach. | Line 52 – 69, page 2: Explanation of the need to explore new marine restoration techniques.  Line 84 – 111, page 3: Explanation of what the Mineral Accretion Technique (MAT) is, and how it works.  Line 112 – 126, page 3: summary on the fragmentation of terminology and information related to MATs in the scientific literature, as well as an explanation of the need to synthesize all the data into a single literature review. |
| Objectives | 4 | Provide an explicit statement of the questions and objectives being addressed with reference to their key elements (e.g., population or participants, concepts, and context) or other relevant key elements used to conceptualize the review questions and/or objectives. | Line 127 – 135, page 4 |
| **METHODS** | | | |
| Protocol and registration | 5 | Indicate whether a review protocol exists; state if and where it can be accessed (e.g., a Web address); and if available, provide registration information, including the registration number. | Line 138, page 4: PRISMA guidelines (referenced in the manuscript). Available at: <https://www.acpjournals.org/doi/10.7326/M18-0850>  Line 150, page 4: Guidelines and Standards for Evidence Synthesis in Environmental Management (referenced in the manuscript). Available at: <https://environmentalevidence.org/information-for-authors/> |
| Eligibility criteria | 6 | Specify characteristics of the sources of evidence used as eligibility criteria (e.g., years considered, language, and publication status), and provide a rationale. | Lines 140 – 147, page 4: sources included any scientific publications due to the small number of sources. |
| Information sources* | 7 | Describe all information sources in the search (e.g., databases with dates of coverage and contact with authors to identify additional sources), as well as the date the most recent search was executed. | Table S1: to check the entire search strategy and selection of our population, intervention, and outcome arguments. It contains all the information on databases, dates, and the number of publications retrieved per search. |
| Search | 8 | Present the full electronic search strategy for at least 1 database, including any limits used, such that it could be repeated. | Text S1: due to the length of the search string in both WOS and Scopus, we decided to include it as a supplemental material. The entire search string for these databases is included in Text S1. |
| Selection of sources of evidence† | 9 | State the process for selecting sources of evidence (i.e., screening and eligibility) included in the scoping review. | Line 154 – 162, page 4. |
| Data charting process‡ | 10 | Describe the methods of charting data from the included sources of evidence (e.g., calibrated forms or forms that have been tested by the team before their use, and whether data charting was done independently or in duplicate) and any processes for obtaining and confirming data from investigators. | Line 155, page 4.  Line 176 – 178, page 5. |
| Data items | 11 | List and define all variables for which data were sought and any assumptions and simplifications made. | Line 168 – 178, page 5. |
| Critical appraisal of individual sources of evidence§ | 12 | If done, provide a rationale for conducting a critical appraisal of included sources of evidence; describe the methods used and how this information was used in any data synthesis (if appropriate). | Line 182 – 190, page 5: a key aspect of the present scoping review is the critical review of the methodology of the reviewed publications due to the lack of consistent and rigorous methodology as highlighted through the manuscript. |
| Synthesis of results | 13 | Describe the methods of handling and summarizing the data that were charted. | Line 179 – 181, page 5: data analysis.  Line 182 – 190, page 5: description of the appliance of the critical appraisal. |
| **RESULTS** | | | |
| Selection of sources of evidence | 14 | Give numbers of sources of evidence screened, assessed for eligibility, and included in the review, with reasons for exclusions at each stage, ideally using a flow diagram. | Line 152 – 162, page 4: included as part of the Methods section since it was more appropriate as a way to describe the methodology used. |
| Characteristics of sources of evidence | 15 | For each source of evidence, present characteristics for which data were charted and provide the citations. | Data S1, “extraction” sheet: it includes all the information extracted from each source. |
| Critical appraisal within sources of evidence | 16 | If done, present data on critical appraisal of included sources of evidence (see item 12). | Line 243, page 7 (table 3) |
| Results of individual sources of evidence | 17 | For each included source of evidence, present the relevant data that were charted that relate to the review questions and objectives. | Data S1, “Keywords” sheet: include all the relevant data for mapping the research landscape related to MATs (objective i: consolidate the existing scientific literature on MATs).  Data S1, “Quality Assessment” sheet or Table 3: objective ii: analyze and evaluate experimental applications of MATs as a benthic restoration tool.  Data S1, “Synthesis” sheet: include all the relevant information related to the current knowledge on MATs effect on benthic organisms (objective iii: synthesize current knowledge on their effects on benthic organism health and recovery potential). |
| Synthesis of results | 18 | Summarize and/or present the charting results as they relate to the review questions and objectives. | Line 194 - 213, page 5: synthesis of results related to objective i.  Line 216 – 239, page 6: synthesis of results related to objective iii.  Line 242 – 351, pages 6 – 9: includes all the synthesis of results related to objective ii. The extension is due to grouping the results into the different themes of the quality assessment framework. |
| **DISCUSSION** | | | |
| Summary of evidence | 19 | Summarize the main results (including an overview of concepts, themes, and types of evidence available), link to the review questions and objectives, and consider the relevance to key groups. | Line 355 – 389, page 9: related to objective i. It summarizes the results according to the current research landscape of MATs and the need for standardization of its terminology.  Line 392 – 424, page 10: related to objective iii. Summary of the current knowledge on the effects of MATs on benthic organisms.  Line 427 – 524, page 11 – 14: related to objective ii. These pages are related to the potential sources of bias shaping the current knowledge of MATs as a benthic restoration tool found during the quality assessment. The findings are grouped by thematic themes: lack of proper control groups (line 426, page 11); methodological replicability of the treatments (line 472, page 12); and deficiencies in statistical reporting (line 511, page 13). |
| Limitations | 20 | Discuss the limitations of the scoping review process. | Line 163 – 165, page 5: despite being presented in the Methods section, it highlights the low number of retrieved sources found and the need for cross-referencing in the scoping process.  Line 357-363, page 9.  Line 378, page 10.  Line 422, page 11. |
| Conclusions | 21 | Provide a general interpretation of the results with respect to the review questions and objectives, as well as potential implications and/or next steps. | Line 551 - 581, page 14. |
| **FUNDING** | | | |
| Funding | 22 | Describe sources of funding for the included sources of evidence, as well as sources of funding for the scoping review. Describe the role of the funders of the scoping review. | Funding Statement, page 1 of generated PDF(not included in the manuscript) |

JBI = Joanna Briggs Institute; PRISMA-ScR = Preferred Reporting Items for Systematic reviews and Meta-Analyses extension for Scoping Reviews.

* Where *sources of evidence* (see second footnote) are compiled from, such as bibliographic databases, social media platforms, and Web sites.

† A more inclusive/heterogeneous term used to account for the different types of evidence or data sources (e.g., quantitative and/or qualitative research, expert opinion, and policy documents) that may be eligible in a scoping review as opposed to only studies. This is not to be confused with *information sources* (see first footnote).

‡ The frameworks by Arksey and O’Malley (6) and Levac and colleagues (7) and the JBI guidance (4, 5) refer to the process of data extraction in a scoping review as data charting*.*

§ The process of systematically examining research evidence to assess its validity, results, and relevance before using it to inform a decision. This term is used for items 12 and 19 instead of "risk of bias" (which is more applicable to systematic reviews of interventions) to include and acknowledge the various sources of evidence that may be used in a scoping review (e.g., quantitative and/or qualitative research, expert opinion, and policy document).

*From:* Tricco AC, Lillie E, Zarin W, O'Brien KK, Colquhoun H, Levac D, et al. PRISMA Extension for Scoping Reviews (PRISMAScR): Checklist and Explanation. Ann Intern Med. 2018;169:467–473. [doi: 10.7326/M18-0850](http://annals.org/aim/fullarticle/2700389/prisma-extension-scoping-reviews-prisma-scr-checklist-explanation).
