# Supplementary material for: Assessing the mineral accretion technique (MAT) for marine benthic restoration: a scoping review highlighting procedural weaknesses and evidence gaps
Source: PeerJ. 2026 Jun 22;14:e21440. doi: 10.7717/peerj.21440 (PMC13296807; doi:10.7717/peerj.21440)
Supplement: Supplemental Information 5 [file peerj-14-21440-s005.docx]

**Text S1. Search String.**

On Scopus: (restor* OR recover* OR ((habitat* OR marine* OR ecolog* OR environmental* OR ecosystem* OR communit* OR " ecosystem AND based " OR active OR passive OR assist*) AND (restor* OR recover* OR transplant* OR transl* OR protect* OR rehabilit* OR management)) OR "restoration AND techn*" OR transpl* OR translocat* OR growth OR survival OR reproduct* OR branch*) AND ("mineral accretion techn*" OR "electric* reef*" OR "electric* artificial reef*" OR biorock OR "biorock technolog*" OR "seawater electrolysis" OR electrolysis OR electrodeposition OR seacrete OR seament OR "mineral deposition" OR "mineral accretion" OR "mineral electrodeposition" OR "low voltage mineral deposition" OR "low-voltage mineral deposition" OR "electrochemical deposition" OR "low voltage mineral accretion" OR "low-voltage mineral accretion") AND (benth* OR seafloor* OR coral* OR "hard bottom" OR "soft bottom" OR subtidal OR "Deep Sea" OR "Deep-Sea" OR mesophotic* OR maërl OR spong* OR seagrass* OR "seagrass meadow*" OR seabed OR reef* OR "rocky reef*" OR anthozoa* OR oyster* OR "oyster reef*" OR "animal forest*" OR "octocoral*").

On WOS: (Topic) restor* OR recover* OR ((habitat* OR marine* OR ecolog* OR environmental* OR ecosystem* OR communit* OR "ecosystem AND based " OR active OR passive OR assist*) AND (restor* OR recover* OR transplant* OR transl* OR protect* OR rehabilit* OR management)) OR "restoration AND techn* " OR transpl* OR translocat* OR growth OR survival OR reproduct* OR branch* (Topic) and "mineral accretion techn*" OR "electric* reef*" OR "electric* artificial reef*" OR biorock OR "biorock technolog*" OR "seawater electrolysis" OR electrolysis OR electrodeposition OR seacrete OR seament OR "mineral deposition" OR "mineral accretion" OR "mineral electrodeposition" OR "low voltage mineral deposition" OR "low-voltage mineral deposition" OR "electrochemical deposition" OR "low voltage mineral accretion" OR "low-voltage mineral accretion" (Topic) and benth* OR seafloor* OR coral* OR "hard bottom" OR "soft bottom" OR subtidal OR "Deep Sea" OR "Deep-Sea" OR mesophotic* OR maërl OR spong* OR seagrass* OR "seagrass meadow*" OR seabed OR reef* OR "rocky reef*" OR anthozoa* OR oyster* OR "oyster reef*" OR "animal forest*" OR "octocoral*"
